# Sphingolipids and Inositol Phosphates Regulate the Tau Protein Phosphorylation Status in Humanized Yeast

**DOI:** 10.3389/fcell.2020.592159

**Published:** 2020-11-17

**Authors:** Francisca Randez-Gil, Lino Bojunga, Francisco Estruch, Joris Winderickx, Maurizio Del Poeta, Jose A. Prieto

**Affiliations:** ^1^Department of Biotechnology, Instituto de Agroquímica y Tecnología de los Alimentos, Consejo Superior de Investigaciones Científicas, Valencia, Spain; ^2^Departament of Biochemistry and Molecular Biology, Universitat de València, Valencia, Spain; ^3^Functional Biology, KU Leuven, Leuven, Belgium; ^4^Department of Molecular Genetics and Microbiology, Stony Brook University, Stony Brook, NY, United States; ^5^Veterans Administration Medical Center, Northport, NY, United States

**Keywords:** *Saccharomyces cerevisiae*, 1-IP_7_, Pho85, ceramide, Sit4, Ypk1, Fpk1, 2

## Abstract

Hyperphosphorylation of protein tau is a hallmark of Alzheimer’s disease (AD). Changes in energy and lipid metabolism have been correlated with the late onset of this neurological disorder. However, it is uncertain if metabolic dysregulation is a consequence of AD or one of the initiating factors of AD pathophysiology. Also, it is unclear whether variations in lipid metabolism regulate the phosphorylation state of tau. Here, we show that in humanized yeast, tau hyperphosphorylation is stimulated by glucose starvation in coincidence with the downregulation of Pho85, the yeast ortholog of CDK5. Changes in inositol phosphate (IP) signaling, which has a central role in energy metabolism, altered tau phosphorylation. Lack of inositol hexakisphosphate kinases Kcs1 and Vip1 (IP_6_ and IP_7_ kinases in mammals) increased tau hyperphosphorylation. Similar effects were found by mutation of *IPK2* (inositol polyphosphate multikinase), or *PLC1*, the yeast phospholipase C gene. These effects may be explained by IP-mediated regulation of Pho85. Indeed, this appeared to be the case for *plc1*, *ipk2*, and *kcs1*. However, the effects of Vip1 on tau phosphorylation were independent of the presence of Pho85, suggesting additional mechanisms. Interestingly, *kcs1* and *vip1* strains, like *pho85*, displayed dysregulated sphingolipid (SL) metabolism. Moreover, genetic and pharmacological inhibition of SL biosynthesis stimulated the appearance of hyperphosphorylated forms of tau, while increased flux through the pathway reduced its abundance. Finally, we demonstrated that Sit4, the yeast ortholog of human PP2A protein phosphatase, is a downstream effector of SL signaling in mediating the tau phosphorylation state. Altogether, our results add new knowledge on the molecular effectors involved in tauopathies and identify new targets for pharmacological intervention.

## Introduction

Alzheimer’s disease (AD) is the most prevalent neurodegenerative disease being responsible for 60–70% of the 50 million cases of dementia every year (Prince et al., 2015). Current treatments for AD remain supportive, without changing the life expectancy or overall progression of dementia (Weller and Budson, 2018). Accordingly, understanding the molecular mechanisms that finally result in this neurodegenerative disorder has been and remains of major importance.

The neurodegenerative process in AD is characterized by the progressive accumulation of amyloid plaques, mainly composed of amyloid-beta peptides (Aβ), and neurofibrillary tangles (NFTs) composed of hyperphosphorylated tau (Jeong, 2017). The amyloid plaques accumulate extracellularly in the brains of AD patients and are generated by sequential processing of the amyloid precursor protein, APP (Glenner et al., 1984; Dyrks et al., 1988). The neurofibrillary tangles are found inside neurons and consist of an abnormally phosphorylated form of the tau protein (Buée et al., 2000). Although Aβ and tau exert their neurotoxic effects through separate mechanisms (Grimm and Hartmann, 2012), several studies suggest that tau is an essential executor of β-amyloid neurotoxicity (Rapoport et al., 2002; Roberson et al., 2007; Ittner et al., 2010). Thus, reducing self-assembled hyperphosphorylated tau might be pivotal to alleviate tau deposition and confer resistance to Aβ-mediated neurodegeneration.

The tau protein belongs to the family of microtubule (MT)-associated proteins (MAPs) (Weingarten et al., 1975). As a result of alternative splicing, six major isoforms are present in human brain (Ksiezak-Reding et al., 1988), which in turn, exhibit different post-translational modifications. Phosphorylation of tau is the most prevalent, occurring in up to 85 amino acid residues (Grimm and Hartmann, 2012; Šimić et al., 2016), and these modification impact on the MT-stabilizing properties of tau (Lindwall and Cole, 1984). In particular, the aberrant phosphorylation (hyperphosphorylation) on several residues (e.g., Thr^31^, Thr^181^, Ser^202^, Ser^205^, Ser^214^, Ser^296^, Ser^404^, Ser^409^, and Ser^422^) severely affects tau’s binding capacity to MT (Šimić et al., 2016).

Several protein kinases, including glycogen synthase kinase GSK3β (Baum et al., 1996; Lucas et al., 2001) and the cyclin-dependent kinase CDK5 (Cruz et al., 2003, 2006; Zheng et al., 2005) are involved in tau phosphorylation (Grimm and Hartmann, 2012; Šimić et al., 2016). Studies have also identified serine/threonine protein phosphatases, in particular PP1 and PP2A, as being important for tau dephosphorylation in mammalian cells (Hernández et al., 2010). CDK5 acts as a modulator of tau hyperphosphorylation via the inhibitory regulation of GSK3β (Plattner et al., 2006; Chow et al., 2014). Furthermore, activated CDK5 promotes tau phosphorylation by inactivating PP1 (Lu et al., 2011). Remarkably, evidence also exists that tau phosphorylation responds to a sequential mechanism, and that tau phosphorylation by GSK3β may favor phosphoepitopes affected by other kinases (Zheng-Fischhöfer et al., 1998). Thus, abnormal increased CDK5 activity contributes to the neurodegenerative process directly and indirectly. However, the mechanisms and effectors involved in regulating CDK5 remain unclear.

It is well established that metabolic dysfunctions contribute to the risk and pathophysiology of neurodegenerative diseases, including the AD (Isacson et al., 2019). Biochemical evidence has established a link between Aβ formation, neuronal death, and alterations in the sphingolipid (SL) metabolism (Alaamery et al., 2020; Crivelli et al., 2020). Plasma SLs have been proposed as diagnostic and prognostic biomarkers of AD (Han et al., 2011) based on the idea of a link between SL changes and progression of cognitive decline in AD patients (Mielke and Haughey, 2012; Couttas et al., 2014). Increased levels of ceramides, triggered by upregulation of ceramide synthase- and sphingomyelinase-encoding genes (Katsel et al., 2007; He et al., 2010) and lower contents of sphingomyelin and sphingosine-1-phosphate (S1P), have been found in AD patients (Han et al., 2002; He et al., 2010; Filippov et al., 2012; van Echten-Deckert and Walter, 2012; Couttas et al., 2014). Alterations in ceramide levels have been postulated to contribute to the apoptotic signaling and favor Aβ formation (Hernández-Corbacho et al., 2017), while low S1P levels would result in a reduction of neuroprotective signals (Moruno-Manchon et al., 2015). However, the discrepancies between different studies on changes in SL metabolism in AD are remarkable (Crivelli et al., 2020). Intriguingly, studies on sphingosine-kinase and sphingosine-degrading enzymes have shown that lowering S1P decreases the Aβ production *in vitro* and *in vivo* (Takasugi et al., 2011; Karaca et al., 2014; Lei et al., 2019). In addition, the relation between tau and the SL metabolism is poorly characterized, and there is no evidence of the implication of these lipids in its regulation.

The Pho80-Pho85 cyclin/cyclin-dependent kinase (CDK) complex is a key component of the phosphate (PHO) signaling response in yeast (Lee et al., 2008). In addition, Pho85, the *Saccharomyces cerevisiae* ortholog of CDK5 (Nishizawa et al., 1999), can bind different cyclins and as such has a pleiotropic role on several aspects of the yeast metabolism (Conrad et al., 2014), including SL biosynthesis. Pho85 modulates the abundance of Ypk1 and Orm2 proteins (Prieto et al., 2020), the regulatory circuit that controls the synthesis of long-chain bases (LCBs) (Liu et al., 2012; Gururaj et al., 2013; Teixeira and Costa, 2016; Roelants et al., 2017), which are the direct precursors of ceramides and complex yeast SLs (Megyeri et al., 2016). Pho85 functions in the phosphorylation and degradation of Lcb4, the major LCB kinase (Iwaki et al., 2005) that plays a role in regulating the steady-state level of yeast-phosphorylated LCBs (LCBPs), i.e., dihydrosphingosine-1-phosphate (DhS1P) and phytosphingosine-1-phosphate (PhS1P). Thus, changes in SL species could mediate the signaling activity of Pho85 and determine the functionality of downstream effectors. Whether such regulatory links exist for CDK5 to control the phosphorylation state of tau in mammalian cells is an open question.

Pho85 is a key element in connecting phosphate metabolism and SL synthesis. When yeast cells are starved of inorganic phosphate, the Pho80-Pho85 cyclin-CDK complex is inactivated by the reversible binding of the diphosphoinositol phosphate (DPIP) 1-IP_7_ which leads to the subsequent transcription of *PHO* genes (Lee et al., 2007, 2008). Likewise, changes in DPIP levels caused by membrane stress, i.e., cold shock, inhibit Pho85 activity leading to a significant change in the levels of LCBs and LCBPs (Prieto et al., 2020). DPIPs, also known as inositol pyrophosphates (Wundenberg and Mayr, 2012), are produced by the sequential action of inositol multiphosphate kinases, Ipk2 and Ipk1, which transform the precursor myo-D-inositol 1, 4, 5-trisphosphate (IP_3_) in myo-inositol 1, 2, 3, 4, 5, 6-hexakisphosphate (IP_6_). Then, IP_6_ is converted into the DPIP 1-IP_7_ or 5-IP_7_ by the action of two IP_6_ kinases, Vip1 (1PP-IP_5_ kinase or IP_7_ kinase in mammals) and Kcs1 (5PP-IP_5_ or IP_6_ kinase in mammals) (Dubois et al., 2002; Mulugu et al., 2007; Lin et al., 2009; Wilson et al., 2013). Interestingly, a loss of inositol polyphosphate multikinase (IPMK) activity has been reported in the striatum of Huntington’s disease (HD) patients and in several cellular and animal models of the disease (Ahmed et al., 2015). Moreover, a link between increased production of 1-IP_7_ and AD has been proposed by tagging single nucleotide polymorphism (SNP) analysis of the *IP6K3* gene, one of the three mammalian alleles encoding IP_6_ kinases, in patients with familial and sporadic late-onset Alzheimer’s disease (LOAD) (Crocco et al., 2016). Nevertheless, the relevance of DPIPs in the context of AD and whether their function is mediated by CDKs need to be addressed.

In this paper, we have taken advantage of the use of humanized yeast to examine the role of IPs and SLs in regulating the phosphorylation state of human tau. Different studies have demonstrated that the budding yeast *S. cerevisiae* is a useful model system for functional studies of human proteins, including those involved in neurodegenerative diseases that do not have a homolog in yeast (Winderickx et al., 2008; Verduyckt et al., 2016; Fruhmann et al., 2017; Tenreiro et al., 2017). More importantly, yeast model of AD recapitulates robustly different important aspects of the tau pathobiology associated with AD pathology, including hyperphosphorylation and aggregation of human tau in a Pho85-dependent manner (Vandebroek et al., 2005, 2006). Finally, yeasts and human cells share most fundamental features of eukaryotic cell biology, and thus, yeast models allow to carrying a screen of an entire genome for discovering mechanisms and effectors involved in protein-misfolding pathologies (Treusch et al., 2011). Our results provide evidence by genetic and pharmacological inhibition of IP and SL pathways that these lipid molecules are implicated in modulating the phosphorylation of tau via Pho85, thereby connecting different lipid signaling pathways to the control of the hallmark protein of tauopathies.

## Materials and Methods

### Strains, Plasmids, Media, and Culture Conditions

The yeast strains, plasmids, and oligonucleotides used in this study are listed in Supplementary Tables S1–S3, respectively. *LCB4* and *LCB5* deletions were carried out by PCR-based gene disruption using plasmid pAG32 (Goldstein and McCusker, 1999) as a template and the appropriate target-gene-specific oligonucleotide pairs (Supplementary Table S2). Pho85 and Pho81 C-terminal tagging with the 13xMyc or 3xHA epitopes was carried out by PCR-based gene tagging using plasmids pFA6a-13Myc-His3MX6 or pFA6a-3HA-His3MX6, respectively (Longtine et al., 1998). Previously described standard methods were used for YPD and SD media preparation (Guthrie and Fink, 1991). SCD contained 2% glucose as a carbon source and the appropriate amino acid dropout mixture (Formedium, England). For drug treatments, phytosphingosine (PhS) (Cayman, Ann Arbor, MI), myriocin (Myr) (Cayman, Ann Arbor, MI), or aureobasidin A (AbA) (Takara, Mountain View, CA) were added to cells grown to the mid-log phase (Abs_600_ ∼0.5) at 25, 2, or 0.068 μM, respectively, and cultures were maintained under shaking at 30°C for 30 min (PhS) or 1 h (Myr and AbA). Stock solutions of 25 mM PhS (methanol), 2 mM Myr (ethanol:DMSO, 80:20, v:v), and 90 mM AbA (ethanol), were prepared, sampled in small volumes, and stored at −20°C until use at the indicated concentrations.

For plate phenotype experiments, cells were grown to the mid-exponential phase at 30°C (Abs_600_ ∼0.5). Then, 10-fold serial dilutions were prepared and 3 μl aliquots of each sample (1–10^–3^) were applied over the agar-gelled plates. Colony growth was inspected after 2–4 days of incubation at 30°C. In some experiments, the growth of yeast transformants was followed in liquid medium by using a POLARstar Omega microplate reader (BMG LABTECH, Germany). For this, 30°C-grown saturated SCD-Ura cultures were diluted in the same medium at initial OD_600_ ∼0.05 and microplate wells were filled with 200 μl. All assays were performed in triplicate at 30°C for 24 h. The growth of yeast mutants in YPD lacking or containing 0.034 or 0.068 μM AbA was followed under the same conditions.

To evaluate the effects of glucose concentration on tau phosphorylation, yeast cells were grown on SCD-Ura to the mid-exponential phase at 30°C (Abs_600_ ∼0.3). At this time, an aliquot of the culture was processed (C0) and the rest was centrifuged. Cells were washed twice in sterile cold water, resuspended in fresh SCD-Ura medium (control culture) or in the same medium containing 0.01% glucose as sole carbon source, and cultured under shaking conditions at 30°C, and aliquots were withdrawn after 1–3 h. In all cases, cells were seeded by centrifugation and washed and the pellet of cells were kept at −80°C until use.

### Preparation of Protein Extracts and Western Blot Analysis

Protein extracts were prepared and proteins were separated by SDS-PAGE and analyzed by a Western blot as formerly described (Córcoles-Sáez et al., 2016). Recombinant tau and phosphorylated (S^396^/S^404^) tau were detected using mouse monoclonal antibodies Tau5 (1:2,000, cat# sc-58,860; Santa Cruz Biotechnology, Santa Cruz, CA) and AD2 (1:2,000, mAb# 56,484; Bio-Rad Laboratories, Inc., Hercules, CA), respectively. Rabbit polyclonal antibody PG5 (1:1,000, cat# 44-760G; Invitrogen, Camarillo, CA) was used to detect the phospho-S^409^ epitope. The proteins tagged with 3xHA (Pho81-HA) and 13xMyc (Pho85-Myc) were visualized with anti-HA (1:2,000, cat# sc-7,392; Santa Cruz Biotechnology) and anti-Myc (1:2,000, cat# sc-40; Santa Cruz Biotechnology) mouse monoclonal antibodies, respectively. Mouse monoclonal anti-Pgk1 (C-terminal; 1:3,000, cat# SAB1402307; Merck KGaA, Darmstadt, Germany) and anti-G6Pdh antibodies (1:3,000; cat# sc-47724; Santa Cruz Biotechnology) were used to detect phosphoglycerate kinase 1 and glucose-6-phosphate dehydrogenase as loading control, respectively. Horseradish peroxidase-conjugated goat anti-rabbit (1:2,000, cat# 7,074; Cell Signaling, Danvers, MA, United States) or rabbit anti-mouse (1:5,000, cat# P0260; Dako, Carpinteria, CA) were used as secondary antibodies. Blots were done as described elsewhere (Córcoles-Sáez et al., 2016). Imaging of chemiluminescent Western blots was carried out by using an ImageQuant^TM^ LAS 4000 instrument (General electric) in the Snow mode. Band intensities expressed as arbitrary units were determined using ImageJ software and normalized relative to the G6Pdh control.

### Protein Fractionation and Tau Localization

Wild-type, *pho85*, and *vip1* mutant cells expressing native human tau were lysed in ice−cold buffer (50 mM HEPES, pH 7.4, 50 mM NaCl), and the corresponding protein extracts were centrifuged at 500 × *g* for 5 min to remove unbroken cells and glass beads. The clear supernatants were recentrifuged at 17,900 × *g* for 10 min. The supernatant (fraction S) was collected, and the Triton X-100-soluble proteins were extracted by incubating the pellet on ice for 30 min with the same volume of lysis buffer that contained 1% Triton X−100 and 0.001% Na^+^−deoxycholate (Babu et al., 2012). The extract was recentrifuged, and the supernatant (fraction S2) was recovered. Finally, the resulting pellet was resuspended in Laemmli loading buffer, heated at 95°C for 5 min and centrifuged and the supernatant containing mostly lipid raft-associated proteins (fraction S3) was recovered. Likewise, fractions S1 and S2 were mixed with loading buffer and treated as above before protein assay by SDS-PAGE and Western blot was conducted.

### Sphingolipid Extraction and Mass Spectrometry Analysis

The cells (∼100 OD_600_ units) grown in SCD (Abs_600_ ∼0.5–1.0) at 30°C were suspended in 1.5 ml of Mandala extraction buffer (Mandala et al., 1995) and lipid standards (C17-SpH, C17-DhS, SpH-1P, and C8-PhS; Avanti Polar Lipids, Alabaster, AL), and glass beads (1.0 g; acid washed, 0.4 mm diameter) were added. Then, the mixture was vortexed three times for 1 min each time and incubated at 60°C for lipid preparation as described (Singh et al., 2011, 2012).

For LC-MS, lipid samples were dissolved in 150 μl of ammonium formate (1 mM) with 0.2% formic acid in methanol and separated in an Accela HPLC system (Thermo Scientific, San Jose, CA) coupled to a Spectra C8 (Peeke Scientific, Redwood city, CA) HPLC column (150 × 3 mm) and the HESI source of a TSQ Quantum Ultra triple quadrupole mass spectrometer (Thermo Fisher Scientific). LCBs and their phosphorylated forms (LCBPs) were analyzed, and the mass spectrometer was operated as described (Singh et al., 2012). Samples were processed by the Xcalibur 2.2 Quan Browser software (Thermo Fisher Scientific) and exported to Excel for reporting results. The levels of the different lipid species in each sample were normalized to the units of OD_600_ processed. All the data were calculated from at least two biological replicates (±SD).

### Determination of Acid Phosphatase Activity

The phosphatase activity was assayed according to the method described by Neef and Kladde (2003) with slight modifications. Briefly, yeast cells were washed twice with water and resuspended in 0.1 M sodium acetate buffer, pH 4.0 at 4°C. The reaction was started by mixing 250 μl of cell suspension, previously incubated at 30°C for 5 min, and 250 μl of 20 mM *p*-nitrophenylphosphate (Merck KGaA, Darmstadt, Germany Sigma-). After 10 min at 30°C, the reaction was stopped by adding 125 μl of 1 M Na_2_CO_3_ and quantified by measuring the absorbance at 420 nm. Activities are reported in units per OD_600_ [(OD_420_)/(OD_600_ × volume (in ml) of cells assayed × 10 min)] and are the mean (±SD) of at least three independent experiments.

### Statistical Analysis

Sample averages were compared using a Student’s *t*-test with the Excel software (Microsoft). *p* < 0.05 (^∗^,^#^) was considered statistically significant.

## Results and Discussion

### The Phosphorylation of Tau Depends on the Cell’s Metabolic State

**FIGURE 1:**
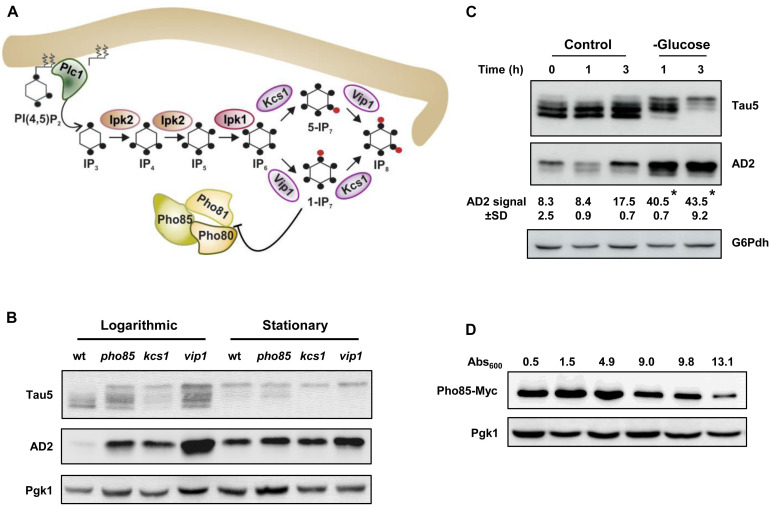
Glucose availability, Pho85, and inositol hexakisphosphate kinases influence tau phosphorylation. **(A)** Schematic representation of the inositol phosphate (IP) pathway and its interaction with the Pho80-Pho81-Pho85 complex. The metabolic steps and enzymes involved in the synthesis of IPs and diphosphoinositol phosphates (DPIPs) are shown (see Dubois et al., 2002; Mulugu et al., 2007; Lin et al., 2009; Wilson et al., 2013 for representative reviews). The hydrolysis of PI(4, 5)P_2_ by Plc1 generates IP_3_, which is sequentially phosphorylated to form in the last steps the DPIPs 1-PP-IP5 (1-IP_7_) and 5-PP-IP5 (5-IP_7_) through the action of the inositol hexakisphosphate kinases Vip1 and Kcs1 (IP_7_ and IP_6_ kinases, in mammals), respectively. The 1-IP_7_ isomer acts as an inhibitor of the cyclin-regulated kinase complex Pho81-Pho80-Pho85 (Lee et al., 2007), which responds to phosphate availability (Lee et al., 2008). The black dot indicates a single phosphate group. The red dot represents a high-energy phosphate or pyrophosphate. For more details, see text. **(B)** Transformants of the CEN.PK2-1C wild-type (wt), *pho85*, *kcs1*, and *vip1* strains harboring the plasmid pYX212-Tau2N/4R (Vandebroek et al., 2006), which express a native isoform of tau, were grown to the mid-logarithmic phase (Logarithmic) in SCD-Ura medium at 30°C. An aliquot of each culture was withdrawn, and the rest was kept under the same conditions until glucose was exhausted (stationary). Protein extracts were prepared and analyzed by SDS-PAGE and Western blot. Antibodies against human tau sequence 218-225 (Tau5) and phospho-S^396^/S^404^ epitopes (AD2) were used to visualize total and hyperphosphorylated tau, respectively. The abundance of phosphoglycerate kinase 1 (Pgk1) visualized with anti-Pgk1 was used as loading control. **(C)** Cell cultures of pYX212-Tau2N/4R transformants of the CEN.PK2-1C wild-type strain were grown until reaching an Abs_600_ ∼0.3 in SCD-Ura. Then, an aliquot was processed (control, 0 h) and the rest of the culture was centrifuged. Cells were washed, transferred to fresh SCD-Ura medium (control), or to the same medium containing 0.01% glucose (glucose) as sole carbon source, grown at 30°C, and samples were taken at the indicated times for Western blot analysis of tau isoforms. Total and hyperphosphorylated tau were visualized by using Tau5 and AD2 antibodies, respectively. The numbers show the AD2 band intensity (±SD) expressed as arbitrary units, which was determined using ImageJ software and normalized relative to the glucose 6-phosphate dehydrogenase control (G6Pdh). Data are the mean (±SD) of three independent experiments. Statistical significance was determined by using a Student’s *t*-test with the Excel software (Microsoft). The glucose starvation samples (glucose) were significantly different compared with their respective control at the same incubation times (*p < 0.05). **(D)** Pho85-Myc tagged cells of the CEN.PK2-1C wild-type strain were grown at 30°C in SCD-Ura medium, harvested at the indicated Abs_600_ values, and protein extracts were compared for Pho85 abundance by Western blot. An antibody against Myc was used. Representative experiments are shown.

Different studies have established a link between glucose energetic hypometabolism and the pathogenesis of AD (Mosconi et al., 2008; Kapogiannis and Mattson, 2011). Recently, it has also been reported that IP6K3 variants that increase the production of DPIP IP_7_, impact on neuronal energy homeostasis and increase the risk of sporadic LOAD (Crocco et al., 2016). Accordingly, we examined the relationship between tau phosphorylation, energy metabolism, and inositol hexakisphosphate kinases, Kcs1 and Vip1, in our yeast model system using the CEN.PK2-1C genetic background. Figure 1A shows a schematic representation of the metabolic steps and enzymes involved in the synthesis of IPs and DPIPs in *S. cerevisiae* and its relationship with the protein kinase Pho85. First, we analyzed the influence of the growth phase in the electrophoretic pattern of tau. For this, yeast cells were transformed with plasmid pYX212-Tau2N/4R (Vandebroek et al., 2006), which expresses the longest native isoform of tau, and the transformants were then grown in SCD-Ura medium. Samples from both actively growing cultures (Abs_600_ ∼0.5) or at the stationary phase (overnight cultures), when glucose is exhausted, were analyzed. The proteins were obtained by alkaline lysis, separated by SDS-PAGE and further visualized by Western Blotting using Tau5, a monoclonal antibody that recognizes the human tau sequence 218–225 independent of its phosphorylation status, and AD2, which is specific for the phosphorylated S^396^/S^404^ epitopes (Buée-Scherrer et al., 1996), a well-known GSK3β/Mds1 targets (Spittaels et al., 2000; Vandebroek et al., 2005, 2006). Protein samples from wild-type (wt) and *pho85* transformants were used as controls. As shown in Figure 1B, different electrophoretic bands with apparent molecular weights varying from 64 to 68 kDa were mainly detected when protein samples from logarithmic-phase grown cells of the wild-type strain were analyzed using the Tau5 antibody. These represent several phospho-forms of tau. In comparison, samples from the *pho85* mutant showed an additional lower mobility band with an apparent molecular weight of approximately 72 kDa that corresponds to hyperphosphorylated tau (Vandebroek et al., 2005; Vanhelmont et al., 2010). The latter is also readily detected using the AD2 antibody (Figure 1B). It has been described that Pho85/CDK5, exerts its function by inhibiting the downstream kinases Mds1/GSK3β, the reason why deletion of *PHO85* leads to hyperphosphorylation of tau (Vandebroek et al., 2006; Vanhelmont et al., 2010). As expected, logarithmic-grown cells of the *kcs1* mutant, which shows increased levels of 1-IP_7_ (Ye et al., 2013; Córcoles-Sáez et al., 2016) and thus a lower activity of the Pho80-Pho85 kinase complex (Lee et al., 2007), displayed a similar profile of bands as the *pho85* mutant. However, protein extracts from v*ip1* cells, which produce the isomer 5-IP_7_ instead of 1-IP_7_ (see Figure 1A), also exhibited a strong lower mobility band revealed by either Tau5 or AD2 antibodies (Figure 1B). It is worth mentioning that unlike 1-IP_7_, the 5-IP_7_ isomer has no apparent inhibitory effect on the Pho80-Pho85 kinase complex activity (Lee et al., 2007).

Concerning stationary-phase-grown cells, glucose starvation rendered tau more susceptible to hyperphosphorylation (Figure 1B). Indeed, while there was an evident reduction in the overall expression of tau in the overnight cultured wild-type cells as judged from the intensity of the Tau5 signal, both Tau5, and AD2 readily detected the lowest mobility band that corresponds to hyperphosphorylated tau (Figure 1B). Consistently, enhanced tau hyperphosphorylation was also evident in the protein extracts of overnight cultures from the *pho85*, the *kcs1*, and the *vip1* strains (Figure 1B). Moreover, SCD-Ura-grown wild-type cells transferred from high (2%) to low glucose (0.01%) medium recapitulated the changes of tau isoforms observed at the stationary phase of growth (Figure 1C). Finally, the enhanced hyperphosphorylation of tau after the logarithmic-phase yeast growth coincided with the gradual decline of Pho85 abundance, as evidenced by Western blot analysis of wild-type cells harboring a Myc-tagged version of the Pho85 protein kinase (Figure 1D). Hence, these changes could be mediated by energy-dependent regulations of Pho85, and likely other protein kinases, by DPIPs. Notably, the deficit in glucose availability in the humanized yeast model thereby seems to mimic the dysregulation of CDK5 (Wu et al., 2000) and increased tau phosphorylation as seen in aging brain (Kelleher et al., 2007).

### Hyperphosphorylation of Tau Has No Major Effect on Yeast Growth but Changes Its Membrane Association

Previous studies indicated that human tau expressed in yeast cells does not affect the growth rate (Vandebroek et al., 2005). We analyzed whether hyperphosphorylation of tau by lack of Pho85, Kcs1, or Vip1 could contribute to increase tau toxicity in our yeast model. As shown in Supplementary Figure S1, the wild-type, *pho85*, *kcs1*, or *vip1* strains transformed with either pYX212-Tau2N/4R or the empty vector control displayed a similar behavior when growth was examined in liquid SCD-Ura, indicative that the expression of tau has no significant impact on the viability of these strains.

**FIGURE 2:**
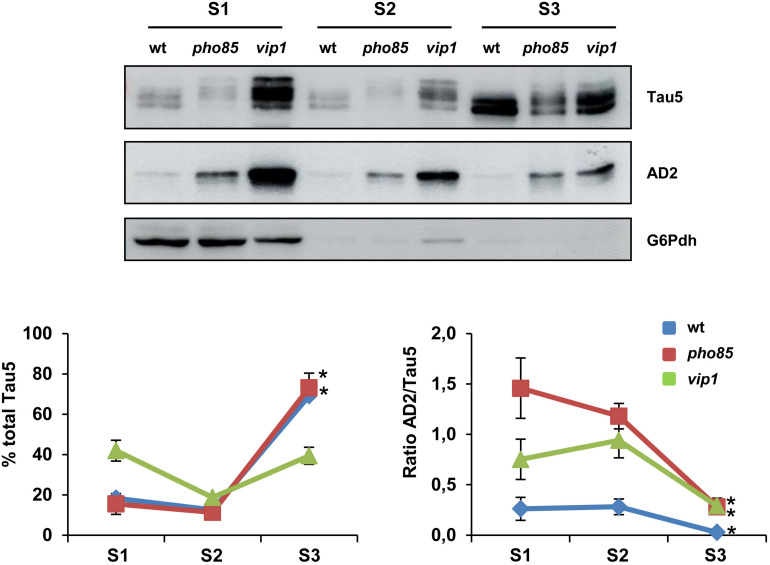
tau localization is influenced by its phosphorylation. Transformants of the CEN.PK2-1C wild-type (wt), *pho85* and *vip1* mutant strains expressing native tau (pYX212-Tau2N/4R) were grown until reaching an Abs_600_ ∼0.3 in SCD-Ura. Protein extracts were processed, fractionated, and analyzed as described in the “Materials and Methods” section. Three fractions, soluble proteins (S1), Triton X-100-soluble proteins (S2), and Triton X-100-insoluble proteins (S3) were analyzed for total and hyperphosphorylated (phospho-S^396^/S^404^ epitopes) tau isoforms by using Tau5 and AD2 antibodies, respectively. The graphs show the relative abundance (%) of total tau (left panel) and the ratio between AD2 and total tau (AD2/Tau5; right panel) for each strain and protein fraction. Band intensities were determined using ImageJ software and normalized relative to the glucose 6-phosphate dehydrogenase control (G6Pdh). Total tau was estimated as the sum of band intensities of tau isoforms with apparent molecular weights varying from 64 to 72 kDa. Data are the mean (±SD) of three independent experiments. Statistical significance (^∗^*p* < 0.05) was determined for S3 sample averages compared with S1 sample averages of each strain. Representative Western blot images are shown.

Next, we analyzed if the enrichment of hyperphosphorylated tau isoforms alters the cellular distribution of the protein in yeast cells. Several studies indicated tau to interact with the plasma membrane and endomembranes (Elbaum-Garfinkle et al., 2010; Georgieva et al., 2014). Moreover, it has been shown that hyperphosphorylation of tau in, among others, S^396^/S^404^ residues completely abolish tau’s association with the membrane (Maas et al., 2000). We examined the electrophoretic profile of tau in soluble and membrane-enriched fractions of crude protein extracts from logarithmic-grown wild-type, *pho85*, and *vip1* cells. Three fractions, corresponding to cytosolic soluble proteins (S1), Triton X-100-soluble proteins (S2), and Triton X-100-insoluble proteins (S3), were analyzed by SDS-PAGE and Western blot (Figure 2). As shown, the profile of bands observed with Tau5 and AD2 antibodies differed between these fractions. The tau protein was mainly localized in the yeast plasma membrane, and in particular in the Triton X-100-insoluble fraction (S3), which was solubilized with SDS (Figure 2; left graph). Only in the *vip1* strain, the soluble S1 fraction contained a higher amount of total tau, which is likely due to hyperphosphorylation as revealed in the AD2 signal (Figure 2). Indeed, the ratio of AD2/Tau5 signals (Figure 2; right graph) clearly indicates that phosphorylation of tau at its AD2 epitope decreases the association of the protein with the yeast plasma membrane.

### Pho85 and Inositol Hexakisphosphate Kinases Influence the Phosphorylation of Tau-S^409^

**FIGURE 3:**
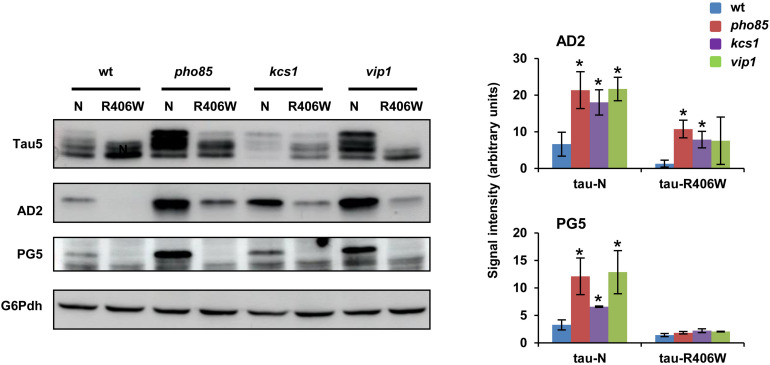
Pho85, Kcs1 and Vip1 contribute to tau phosphorylation by different kinases. Native wild-type tau (N) and the clinical R406W tau mutant (Vanhelmont et al., 2010) were expressed in wild-type (wt), *pho85*, *kcs1*, and *vip1* strains, and the electrophoretic profile of the protein from logarithmic-SCD-Ura-grown (Abs_600_ ∼0.3) cells was analyzed by Western blot using Tau5 and phospho-specific antibodies against S^396^/S^404^ (AD2) and S^409^ (PG5) epitopes. The graphs show the AD2 and PG5 band intensities (±SD) expressed as arbitrary units. Protein extraction, band intensity determination, and statistical analysis were as described in Figure 1C. Data are the mean (±SD) of three independent experiments. *p < 0.05 for AD2 or PG5 band intensity of the mutant strains compared with AD2 or PG5 band intensity of the wild-type control strain. Representative Western blots images are shown.

The results described above confirmed the role of Pho85, Kcs1, and Vip1 in regulating the Mds1/GSK3β-dependent phosphorylation of S^396^/S^404^ at the AD2 epitope of tau. At this point, we wondered if this functional interaction extends to other tau kinases. Previous work has demonstrated a link between phosphorylation of S^396^/S^404^ and S^409^, a target of PKA (Terwel et al., 2005; Vandebroek et al., 2006) that is crucial for tau aggregation in yeast (Vanhelmont et al., 2010). Since it was then shown that the clinical mutant tau-R406W is hampered for phosphorylation S^396^/S^404^ and S^409^, we also expressed this clinical tau mutant in the wild-type, *pho85*, *kcs1*, and *vip1* strains. Western blot analysis confirmed that the 72-kDa low mobility band as visualized with either Tau5 or more clearly with the AD2 antibody is only weakly present upon expression of native wild-type tau but completely absent upon expression of the mutant tau-R406W in wild-type cells (Figure 3). However, the lack of Pho85 activity still increased the S^396^/S^404^ hyperphosphorylation of both wild-type tau and tau-R406W (Figure 3). Also, cells lacking Kcs1 or Vip1 displayed higher levels of the hyperphosphorylated 72 kDa isoform upon expression of wild-type tau, although only the mutation of *KCS1* produced a statistically significant positive effect on tau-R409W (Figure 3; AD2 image and graph). To further confirm these results, protein samples from all the abovementioned transformants were prepared again and tau was visualized with PG5, a phospho-specific antibody that recognizes the phospho-S^409^ epitope. As expected, lack of either Pho85, Kcs1, or Vip1 stimulated not only the phosphorylation of S^396^/S^404^ but also of S^409^ (Figure 3; PG5 image and graph). We conclude that Pho85, Kcs1, and Vip1 contribute to tau phosphorylation by different kinases.

### The IP Pathway Plays a Role in Tau Phosphorylation

**FIGURE 4:**
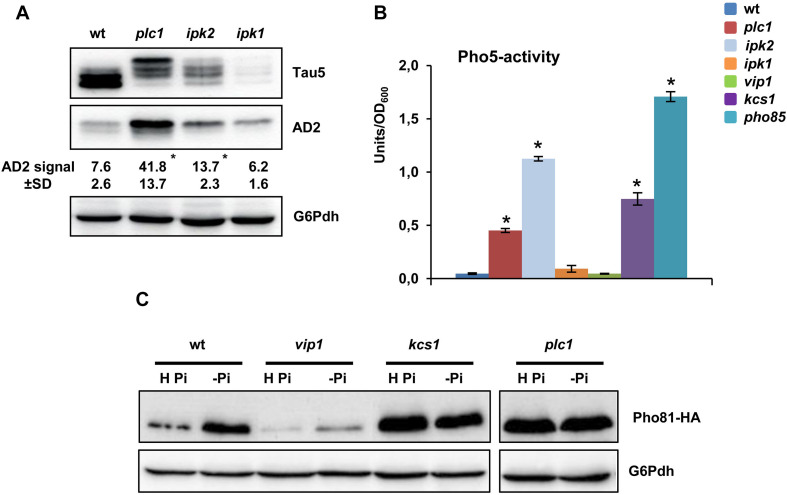
Tau phosphorylation is regulated by IP signaling by Pho85-dependent and Pho85-independent mechanisms. **(A)** Protein extracts from SCD-grown cultures of wild-type (wt), *plc1*, *ipk2*, and *ipk1* transformants of the CEN.PK2-1C yeast background, which express a native isoform of tau, were analyzed by Western blot using Tau5 and AD2 antibodies. The numbers show the AD2 band intensity (±SD) expressed as arbitrary units. Quantification was carried out as in Figure 1C. Data are the mean (±SD) of three independent experiments. *p < 0.05 for AD2 band intensitiy of the mutant strains compared with AD2 band intensity of the wild-type control strain. **(B)** The activity of the Pho85-repressible acid phosphatase Pho5 (units/OD_600_) was measured in protein extracts from logarithmic-SCD-Ura-grown (Abs_600_ ∼0.3–0.5) cultures. Cells of the wild-type (wt), *plc1*, *ipk2*, *ipk1*, *vip1*, *kcs1*, and *pho85* mutants were analyzed. Data represent the mean value (±SD) of three independent experiments. *p < 0.05 for Pho5 activity of the mutant strains compared with the Pho5 activity of the wild-type control strain. **(C)** The abundance of Pho81 in Pho81-HA-tagged cells of the CEN.PK2-1C wild-type, *vip1*, *kcs1*, and *plc1* mutant strains was measured before and after transfer for 3 h of yeast cells from high-phosphate (H Pi) to phosphate-free medium (-Pi). Antibodies against HA and glucose-6-phosphate dehydrogenase (G6Pdh; loading control) were used. A representative experiment is shown.

We next examined whether the role of inositol hexakisphoshate kinases Kcs1 and Vip1 in modulating tau phosphorylation extends to upstream kinases Ipk2 and Ipk1, and Plc1, the yeast phospholipase (Figure 1A). As compared with the wild type, knockout of *IPK1*, which interrupts the IP pathway at the IP_5_ level, decreased the abundance of total tau (Figure 4A; Tau5 image), although its phosphorylation level was not apparently affected (Figure 4A; AD2 image). On the contrary, lack of Ipk2, the enzyme that converts IP_3_ into IP_5_, or *PLC1* that completely abolish IP and DPIP synthesis (Figure 1A), resulted in increased levels of the hyperphosphorylated 72 kDa isoform of tau (Figure 4A).

Then, we analyzed if the effect on tau phosphorylation observed in the *plc1*, *ipk2*, and *vip1* mutants could be explained as a result of the inactivation of Pho85. All of these strains share in common to make no 1-IP_7_, while *kcs1* shows enhanced levels of the DPIP isomer (Ye et al., 2013; Córcoles-Sáez et al., 2016). Nevertheless, IP_4_ and/or IP_5_, and Plc1, also play a role in regulating the transcription of some Pho85-dependent genes, although strain-dependent discrepancies have been reported (Steger et al., 2003; Auesukaree et al., 2005; Prieto et al., 2020). To clarify this point in the CEN.PK2-1C yeast background, we first assayed the activity of the Pho85-repressed acid phosphatase Pho5 in SCD-grown cells (Figure 4B). As expected, Pho5 activity was fully dysregulated in cells of the *pho85* mutant. Compared with this, lack of Kcs1, but also of Ipk2 and Plc1 caused a partial dysregulation. On the contrary, no effect could be detected by deletion of *VIP1* (Figure 4B). Neither the mutation of *IPK1* caused the upregulation of Pho5 activity. To further confirm these results, we compared the abundance of the cyclin-dependent kinase (CDK) inhibitor Pho81, which downregulates the activity of the Pho80-Pho85 complex, in wild-type, *kcs1*, *vip1*, and *plc1* yeast strains. As it is shown in Figure 4C, transfer of wild-type cells from high phosphate to starvation medium stimulated the synthesis of Pho81. As for Pho5 activity, lack of Kcs1 dysregulated the expression of Pho81 and high levels of the protein could be detected even in high-phosphate conditions, a similar result to that observed for *pho85* mutant cells (data not shown). Likewise, the *PLC1* deletion also stimulated the synthesis of Pho81, while *vip1* cells were insensitive to phosphate starvation (Figure 4C). Hence, our results provide evidence of the involvement of the IP pathway in the regulation of tau phosphorylation. Thereby, changes in 1-IP_7_ levels are certainly critical to modulate the activity of Pho85, but additional mechanisms have to be evoked to fully explain the functional link between elements of the IP pathway, like Vip1, and tau phosphorylation.

### Interplay Between Sphingolipids and Tau Hyperphosphorylation

**FIGURE 5:**
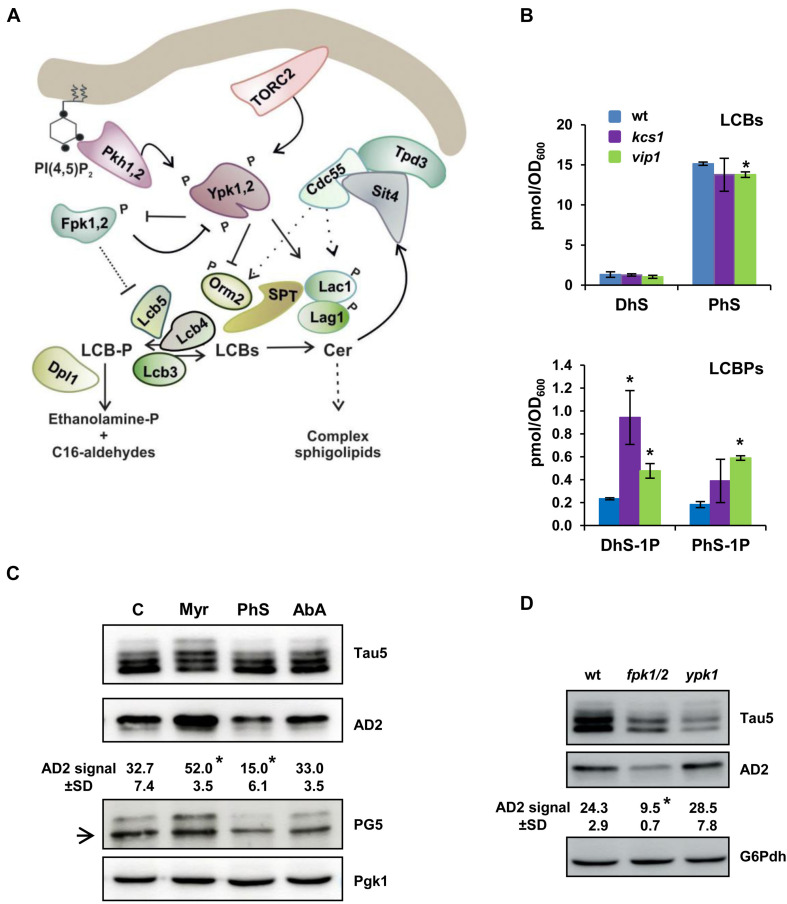
Dysregulated SLs biosynthesis alters the tau electrophoretic profile. **(A)** Schematic representation showing the initial steps of the SLs biosynthesis from the rate-limiting step catalyzed by the serine palmitoyltransferase (SPT) complex to ceramide synthase (Lag1, Lac1), and the LCBs-to-glycerophospholipid metabolic pathway. Orm2 acts as an inhibitor of SPT. LCBs-kinases (Lcb4, Lcb5) use DhS and PhS, the precursors of ceramide (Cer), to form their corresponding phosphorylated forms DhS-1P and PhS-1P. Details about each step, and the enzymes, effectors and regulators involved, can be found in the text and recent reviews (Gururaj et al., 2013; Megyeri et al., 2016; Teixeira and Costa, 2016; Roelants et al., 2017). **(B)** The cells of the CEN.PK2-1C wild-type and its corresponding *kcs1* and *vip1* mutant strains were grown in SCD medium at 30 °C until the mid-logarithmic phase (Abs_600_ ∼0.5). Lipids were extracted, and the LCBs and LCBPs levels were analyzed by mass spectrometry as described in the “Materials and Methods” section. Data are the mean (±SD) of two independent biological replicates. Statistically significant differences (*p < 0.05) between the wild-type and mutant strains are indicated. **(C)** Cells of the CEN.PK2-1C wild-type strain grown in SCD-Ura medium lacking (C) or transferred for 1 h to the same medium containing 2 μM myriocin (Myr) or 0.068 μM aureobasidin A (AbA) were examined for tau isoforms by Western blot of protein extracts. Cells exposed for 30 min to external 25 μM phytosphingosine (PhS) were also analyzed. Tau5, AD2, and PG5 antibodies were used to visualize total and hyperphosphorylated tau, respectively. An inespecific PG5 signal is indicated with an arrow. Quantification of band intensities was carried out as described in Figure 1C. Data represent the mean value (±SD) of three independent experiments. Statistically significant differences (*p < 0.05) between control and treatment samples are indicated. **(D)** The electrophoretic pattern of tau from pYX212-Tau2N/4R transformants of the BY4741 wild-type (wt), *fpk1 fpk2* (*fpk1/2*), and *ypk1* strains grown in SCD-Ura medium were analyzed as in **(C)**. Representative Western blot images are shown.

The IP pathway is networked with other important cellular signaling pathways (Huang and O’Shea, 2005) and lipid metabolic routes (Córcoles-Sáez et al., 2016; Prieto et al., 2020) that could represent additional points of tau regulation. In particular, Pho85 has been identified as playing a key role in controlling the synthesis of LCBs and LCBPs via the Ypk1-Orm2 regulatory circuit (Prieto et al., 2020) and the LCB-kinase Lcb4 (Iwaki et al., 2005; see Figure 5A for a representation of the SL pathway). Thus, *pho85* mutant cells present a unique profile of complex SLs (da Silveira Dos Santos et al., 2014), decreased LCB content, and accumulation of LCBPs (Prieto et al., 2020). These evidences led us to examine the presence of sphingoid bases in the inositol hexakisphosphate kinase mutant *kcs1* and *vip1*. As it is shown in Figure 5B, DhS and PhS levels from *kcs1* and *vip1* mutant cells were almost identical to those of the wild type. Only the amount of PhS was slightly reduced in the *vip1* strain (Figure 5B). Compared with this, both mutants displayed an important variation in their content of phosphorylated sphingoid bases. The DhS-1P level increased fourfold in *kcs1* cells, and remarkably, the amount of DhS-1P and PhS-1P raised two- and threefold, respectively, in the *vip1* mutant (Figure 5B).

Then, we analyzed the electrophoretic profile of tau in cells treated with PhS, the main LCB subclass in yeast (Figure 5B), Myr, or AbA. Myr is an inhibitor of SPT (Miyake et al., 1995), the enzymatic complex that catalyzes the first step in the SL biosynthesis pathway (Hanada, 2003; Figure 5A), and thus, myriocin treatment reduces the carbon flux through the pathway (Han et al., 2010), decreasing the content of LCBs/LCBPs and complex SLs (Huang et al., 2012). AbA treatment inhibits the activity of Aur1, the first enzyme involved in the synthesis of complex SLs, which results in increased amounts of ceramides but lower complex SLs (Nagiec et al., 1997). As shown in Figure 5C, treatment of wild-type yeast cells with AbA had no major effect on the abundance of hyperphosphorylated tau. However, addition of Myr to the yeast culture increased the abundance of hyperphosphorylated tau isoforms visualized with Tau5, AD2, or PG5 antibodies (Figure 5C), indicating that a defective SL pathway stimulates tau phosphorylation. On the contrary, increased LCBs by adding external PhS reduced the abundance of both phospho-S^396^/S^404^ (Figure 5C; AD2) and phospho-S^409^ tau (Figure 5C; PG5).

Finally, we examined the tau phosphorylation profile in yeast mutants lacking kinases involved in the regulation of the SL synthesis. In particular, we paid special attention to the behavior of tau in cells lacking Ypk1 and Fpk1,2. The protein kinase Ypk1 stimulates the SL synthesis by inhibiting the activity of Orm2 (Breslow et al., 2010; Roelants et al., 2011), a negative regulator of SPT (Figure 5A). On the other hand, the homologous protein kinases Fpk1,2 inhibit Ypk1 by phosphorylation, which depresses the SPT activity by increasing the inhibition mediated by Orm2 (Roelants et al., 2010). Thus, lack of Ypk1 and Fpk1,2 reduces and increases, respectively, the SPT-catalyzed synthesis of LCBs and, consequently, the flux through the SL-biosynthesis pathway (Figure 5A). As shown in Figure 5D, the loss of Ypk1 activity increased slightly the hyperphosphorylation of tau as compared with wild-type cells, although the differences were not statistically significant. It is worth noting that Ypk1 share an extensive homology with a redundant protein kinase Ypk2, and that cells lacking both Ypk1 and Ypk2 are inviable (Chen et al., 1993). Contrastingly, the simultaneous disruption of *FPK1* and its homolog *FPK2*, the two protein kinases that mediate the negative regulation of Ypk1 (Figure 5A), caused a clear and significant reduction of tau hyperphosphorylation (Figure 5D). Altogether, our results strongly support a role of the SL metabolism in regulating tau hyperphosphorylation.

### The Ceramide-Regulated Protein Phosphatase Sit4 Plays a Major Role in Tau’s Dephosphorylation

The LCB kinases and phosphatases play an important role in regulating the irreversible degradation of LCBs to ethanolamine-phosphate and C16-aldehydes, and consequently, their activity modulate the carbon flux through the SL biosynthesis pathway (Figure 5A). On the other hand, Sit4, a ceramide-activated protein phosphatase, is the yeast ortholog of human PP2A, which is involved in the dephosphorylation of tau (Sontag and Sontag, 2014; Taleski and Sontag, 2018). Thus, Sit4 could be a key element in connecting SL metabolism and tau hyperphosphorylation, but no evidence of this function in humanized yeast has been reported yet.

**FIGURE 6:**
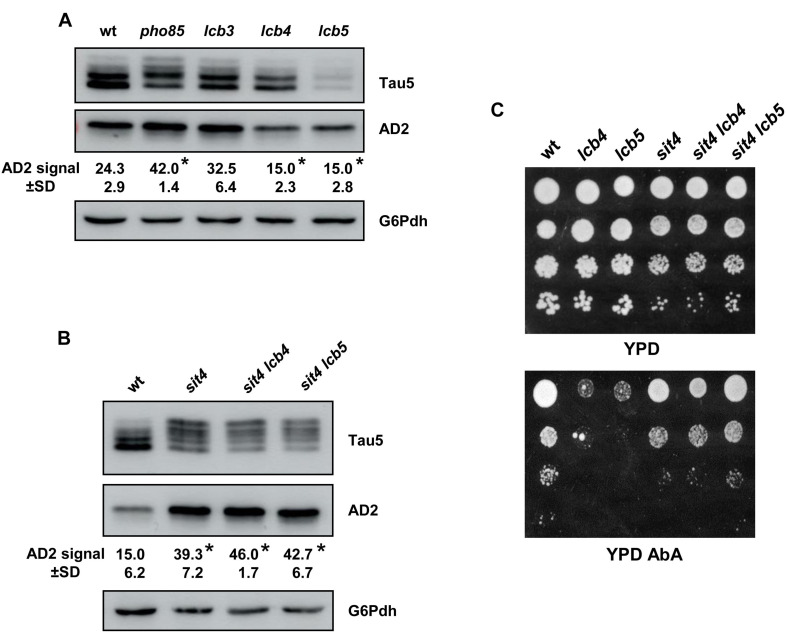
The Sit4 protein phosphatase is a SL-downstream target in regulating tau phosphorylation. **(A)** The tau isoforms from pYX212-Tau2N/4R transformants of the BY4741 wild-type (wt), *pho85*, *lcb3*, *lcb4*, and *lcb5* mutant strains grown in SCD-Ura medium were analyzed by Western blot as described in Figure 1C. Data represent the mean value (±SD) of three independent experiments. Statistically significant differences (*p* < 0.05) between mutant strains and the wild-type control strain are indicated (*). **(B)** Proteins extracts from logarithmic-grown cultures of pYX212-Tau2N/4R transformants of the CEN.PK2-1C wild-type (wt) strain, *sit4*, *sit4 lcb4*, and *sit4 lcb5* were analyzed as in **(A)**. **(C)** Cells of the same strains were examined for growth in YPD lacking or containing 0.068 μM aureobasidin A (YPD+AbA). Overnight YPD-grown cultures were adjusted to Abs_600_ ∼0.5, diluted (1–10^–3^), spotted (3 μl) onto the mentioned media, and incubated at 30°C for 2–5 days. A representative experiment is shown.

Here, we first inspected the profile of the tau phospho-isoforms by visualization with Tau5 and AD2 antibodies in cells of the *lcb3*, *lcb4*, and *lcb5* mutants, using samples from the wild-type and *pho85* strains as control (Figure 6A). Like *pho85* cells, absence of Lcb3-mediated LCBP-phosphatase activity, which favors the degradation of LCBs, increased slightly the intensity of the 72-kDa low mobility tau band visualized with AD2, but the change was not significant. In contrast, reducing LCB-kinase activity by deleting of either *LCB4* and *LCB5* resulted in a reduced abundance of this hyperphosphorylated tau isophorm (Figure 6A).

Then, we examined whether these changes might be related with the activity of Sit4 and whether the mutation of *SIT4* affects the tau phospho-isoform profile. Indeed, protein samples isolated from *sit4* cells appeared to contain a higher fraction of hyperphosphorylated tau (Figure 6B). Interestingly, the additional mutation of *LCB4* or *LCB5* in the *sit4* background, did not affect the tau pattern observed in the *sit4* mutant strain (Figure 6B), indicating that Sit4 is placed downstream of the LCB kinases. To further confirm this point, we checked the phenotype of all these mutants in YPD plates containing AbA (Figure 6C). As indicated, AbA exposure increases the level of ceramides, a well-known inducer of apoptosis and cell death (Hannun and Obeid, 2011), and thus, AbA sensitivity is an indirect way to estimate the cell’s content of this lipid class. As shown, the *lcb4* and *lcb5* single mutant cells exhibited strong sensitivity to the drug, indicating higher ceramide levels than the wild type (Figure 6C). Cells lacking Sit4 activity showed a slight defect of growth, consistent with the multiple regulatory roles of the protein phosphatase in different metabolic processes (Vilaça et al., 2018), but again, the absence of Sit4 supressed the phenotype of *lcb4* and *lcb5* mutant strains (Figure 6C). A positive effect of the lack of Sit4, in particular for the double *sit4 lcb5* mutant, was also observed when the growth was monitored in YPD liquid medium lacking or containing AbA at different concentrations (Supplementary Figure S2).

## Concluding Remarks

In the last years, increasing evidence supports the hypothesis that dysregulated energy and lipid metabolism could be a key mechanism contributing to the pathogenesis of neurodegenerative disorders. Here, we show for the first time that changes in the IP signaling, which act as an energy sensor, promote molecular alterations underlying tauopathies. This is in good agreement with recent research showing that dysregulation of *IP6K* gene contributes to LOAD (Crocco et al., 2016). Defects in this metabolism affect the activity of Pho85, the yeast ortholog of CDK5, which could explain the strong phosphorylation of tau found in some mutants of the pathway, such as *kcs1*. In this context, the finding that avoiding the synthesis of 1-IP_7_ by deletion of *PLC1*, *IPK1*, or *VIP1* also results in tau hyperphosphorylation adds complexity to the tau regulation and suggests the existence of Pho85-independent mechanisms that requires further investigation. A better understanding of the networking operating between the IP pathway and other lipid pathways will support further the development of IP-targeted molecules in therapeutic approaches.

Interestingly, *vip1* and *kcs1* mutant cells showed a strong accumulation of LCBPs, suggesting an aberrant SLs metabolism by dysregulation of the IPs pathway. Consistent with this, we showed by genetic and drug-treatment approaches that inhibiting the SLs pathway stimulates the hyperphosphorylation of tau, while the opposite effect is observed by increasing SLs synthesis. Moreover, we found that Sit4, the yeast ortholog of PP2A and ceramide-activated protein phosphatase, is a downstream target of SLs signaling that impacts on tau phosphorylation. Changes in SLs metabolism are a hallmark of different neurodegenerative disorders, including AD (Alaamery et al., 2020; Crivelli et al., 2020), and drugs targeting S1P and its receptors (O’Sullivan and Dev, 2017) or lowering ceramide levels (Brodowicz et al., 2018) have been claimed as potentially useful for the treatment of AD. However, studies on sphingosine kinase and sphingosine-degrading enzymes have shown that lowering sphingosine kinase 2 (SK2) activity and S1P levels decreases the Aβ production *in vitro* and *in vivo* (Takasugi et al., 2011; Karaca et al., 2014; Lei et al., 2019). In this scenario, our study adds new knowledge on the effectors and molecular events that linked SLs and tau phosphorylation. Taking into account the diversity of SLs functions and the complexity and interdependency of signals they generate, the yeast model can be exploited to speed up the advances in the field of AD and other neurodegenerative diseases as to provide putative novel targets for therapeutic intervention.

## Data Availability Statement

The original contributions presented in the study are included in the article/Supplementary Material, further inquiries can be directed to the corresponding author.

## Author Contributions

FR-G, FE, JW, and JAP designed the study and interpreted the data. FR-G and LB performed most of the experiments. MDP contributed with the analysis of SLs. JAP prepared the manuscript. All authors have read and approved the manuscript.

## Conflict of Interest

JW was co-founder and shareholder of the KU Leuven spin-off companies reMYND nv (Leuven, Belgium) and ADx NeuroSciences nv (Ghent, Belgium). MDP was co-founder and Chief Scientific Officer (CSO) of MicroRid Technologies Inc. (Dix Hills, NY). They declare this did not influence in any way the studies reported in the manuscript. The remaining authors declare that the research was conducted in the absence of any commercial or financial relationships that could be construed as a potential conflict of interest.
