# Supplementary material for: Sphingolipids and Inositol Phosphates Regulate the Tau Protein Phosphorylation Status in Humanized Yeast
Source: Front Cell Dev Biol. 2020 Nov 17;8:592159. doi: 10.3389/fcell.2020.592159 (PMC7705114; doi:10.3389/fcell.2020.592159)
Supplement: Supplementary file 1 [file Table_1.DOCX]

**SUPPLEMENTARY MATERIAL**

**Sphingolipids and inositol phosphates regulate the Tau protein phosphorylation status in humanized yeast**

***Francisca Randez-Gil, Lino Bojunga, Francisco Estruch,***

***Joris Winderickx, Maurizio Del Poeta, and Jose A. Prieto***

**CONTENTS:**

Table S1. *Saccharomyces cerevisiae* strains used in this work

**Table S2.** The plasmids used in this study

**Table S3.** Oligonucleotides used in this study

**FIGURE S1.** tau expression has no major effects on yeast growth

**FIGURE S2.** Sit4 shows a genetic interaction with Lcb4 and Lcb5

**Table S1.** The *Saccharomyces cerevisiae* strains used in this study

| Strain | Genotype | Reference or source |
| --- | --- | --- |
| CEN.PK2-1C | MATa *ura3-52 his3*-Δ*1 leu*-*2*-*3*, *112 trp1*-*289* | M. Rose |
| *pho85* | CEN.PK2-1C *pho85::hphMX4* | (Córcoles-Sáez, *et al*., 2016) |
| *kcs1* | CEN.PK2-1C *kcs1::hphMX4* | (Córcoles-Sáez, *et al*., 2016) |
| *vip1* | CEN.PK2-1C *vip1::hphMX4* | (Córcoles-Sáez, *et al*., 2016) |
| *plc1* | CEN.PK2-1C *plc1::kanMX4* | (Prieto *et al*., 2020) |
| *ipk1* | CEN.PK2-1C *ipk1::hphMX4* | (Córcoles-Sáez, *et al*., 2016) |
| *ipk2* | CEN.PK2-1C *ipk2::hphMX4* | (Córcoles-Sáez, *et al*., 2016) |
| CEN.PK2-1C Pho85-Myc | CEN.PK2-1C *PHO85-*13Myc::*His3MX6* | This study |
| CEN.PK2-1C Pho81-HA | CEN.PK2-1C *PHO81-*3HA::*His3MX6* | This study |
| *kcs1* Pho81-Myc- | *kcs1 PHO81-*3HA::*His3MX6* | This study |
| *vip1* Pho81-Myc | *vip1*Δ *PHO81-*3HA::*His3MX6* | This study |
| *plc1* Pho81-Myc | *plc1*Δ *PHO81-*3HA::*His3MX6* | This study |
| BY4741 | *MATa his3-Δ1 leu2Δ0 met15Δ0 ura3Δ0* | Euroscarf |
| *pho85* | BY4741 *pho85::kanMX4* | Euroscarf |
| *sit4* | BY4741 *sit4::kanMX4* | Euroscarf |
| *lcb3* | BY4741 *lcb3::kanMX4* | Euroscarf |
| *lcb4* | BY4741 *lcb4::kanMX4* | Euroscarf |
| *lcb5* | BY4741 *lcb5::kanMX4* | Euroscarf |
| *sit4 lcb4* | BY4741 *sit4::kanMX4 lcb4::hphMX4* | This study |
| *sit4 lcb5* | BY4741 *sit4::kanMX4 lcb5::hphMX4* | This study |

**Table S2.** The plasmids used in this study

| Plasmid | Description | Source or reference |
| --- | --- | --- |
|  |  |  |
| YEpLac195 | Control plasmid with *URA3* as selection marker | (Gietz and Sugino, 1988) |
| pTau 2N/4R | 2µ plasmid containing the sequence of human Tau 2N/4R under the control of the *TPI1* gene promoter. Selection marker *URA3* | (Vandebroek *et al*., 2006) |
| pTau R406W (2N/4R) | 2µ plasmid containing a clinical mutation of human Tau (Arg 406 changed by Trp) under the control of the *TPI1* gene promoter. Selection marker *URA3* | (Vanhelmont *et al*., 2010) |
| pFA6a-13Myc-His3MX6 | pFA6a-His3MX6-derived plasmid containing sequences encoding 13 tandem repeats of the Myc epitope | (Longtine *et al*., 1998) |
| pFA6a-3HA-His3MX6 | pFA6a-His3MX6-derived plasmid containing sequences encoding 3 tandem repeats of the influenza virus hemagglutinin epitope | (Longtine *et al*., 1998) |
| pAG32 | Cassette amplification template with hphMX4 as selection marker | (Goldstein and McCusker, 1999) |

**Table S3.** The plasmids used in this study

| Name | Sequence (5´-3´) | Use |
| --- | --- | --- |
| LCB4-F1 | GTCTAGCAGCGAAAAGTACGCGAAGAATCTACTATAGATACG  GATCCCCGGGTTAATTAA | Deletion *LCB4* |
| LCB4-R1 | TTACAAAAAAATCATTTTTGAAGGAAAATATAACGTTAATGAATTCGAGCTCGTTTAAAC | Deletion *LCB4* |
| LCB4-V2 | ATTATTACAATACCACCTGG | Deletion verification *LCB4* |
| LCB5-F1 | ATGACTTTGAAACCTTCAAAGAGACGTAAGGGCAGGTCTCCG  GATCCCCGGGTTAATTAA | Deletion *LCB5* |
| LCB5-R1 | GATTAATTGTTCAGTACGAAGGAAAAGATTAAGTAAAGTGGAATTCGAGCTCGTTTAAAC | Deletion *LCB5* |
| LCB5-V2 | TTCAGTTGCCACCGCTTTTC | Deletion verification *LCB5* |
| KANS2 | GTCAAGGAGGGTATTCTGG | Verification integration |
| PHO81-F2 | ACGCTTGTGAGTTGCTTTTTGAGAATAATATTGATATGCGGATCCCCGGGTTAATTAA | Genetic fusion of HA to *PHO81* |
| PHO81-R1 | AAATAATGTATAAGATTTCAAAACTACATATTACAGAACTGAATTCGAGCTCGTTTAAAC | Genetic fusion of HA to *PHO81* |
| PHO81-V2 | GTAACGCTTCCATATGAAG | Verification fusion of HA to *PHO81* |
| PHO85-F2 | GCATCACCCTTGGTTTGCAGAGTACTACCACCACGCTTCACGGATCCCCGGGTTAATTAA | Genetic fusion of Myc to *PHO85* |
| PHO85-R1 | ATATATACATGGCTACGGTTTTTCGCTGACGGGCTGCGGAATTCGAGCTCGTTTAAAC | Genetic fusion of Myc to *PHO85* |
| PHO85-V2 | CAGCAACGACCACCAAGAG | Verification fusion of Myc to *PHO85* |
| Tau-F | AAGATCGGCTCCACTGAGAA | Tau cDNA Amplification |
| Tau-R | GGACGTGGGTGATATTGTCC | Tau cDNA Amplification |
| ACT1-F | GGATCTTCTACTACATCAGC | Actin gene amplification |
| ACT1-R | CACATACCAGAACCGTTATC | Actin gene amplification |
| Oligo dT | TTTTTTTTTTTTTTTTTTC/G/A | cDNA synthesis |

**
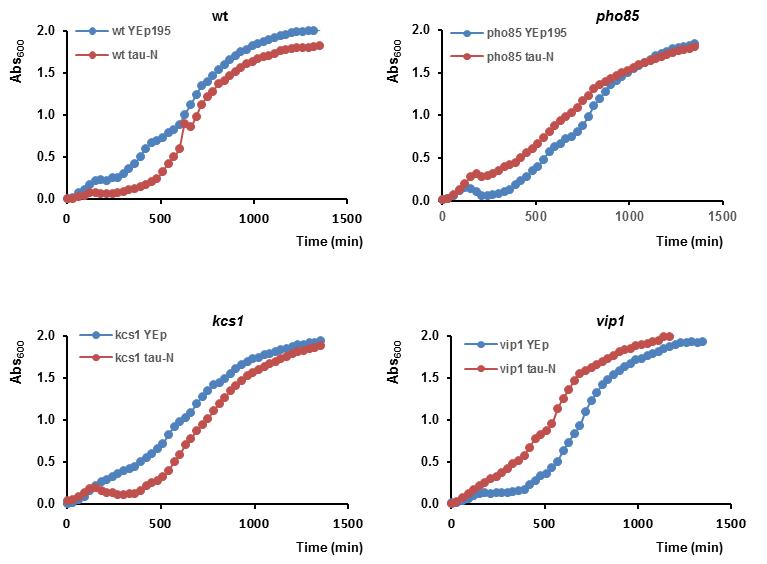
FIGURE S1**. tau expression has no major effects on yeast growth. Saturated cultures of CEN.PK2-1C transformants of the wild-type (wt), *pho85*, *kcs1* and *vip1* mutant strains harbouring plasmid pYX212-Tau2N/4R (Vandebroek et al., 2006), which expresses the longest native isoform of tau (tau-N), were diluted in fresh SCD-medium (Abs_600_ = 0.05) and their growth was recorded by using a POLARstar Omega microplate reader. All assays were performed in triplicate at 30°C for 24 h. Growth curves of yeast cells transformed with an empty plasmid (YEplac195; YEp) were used as controls.


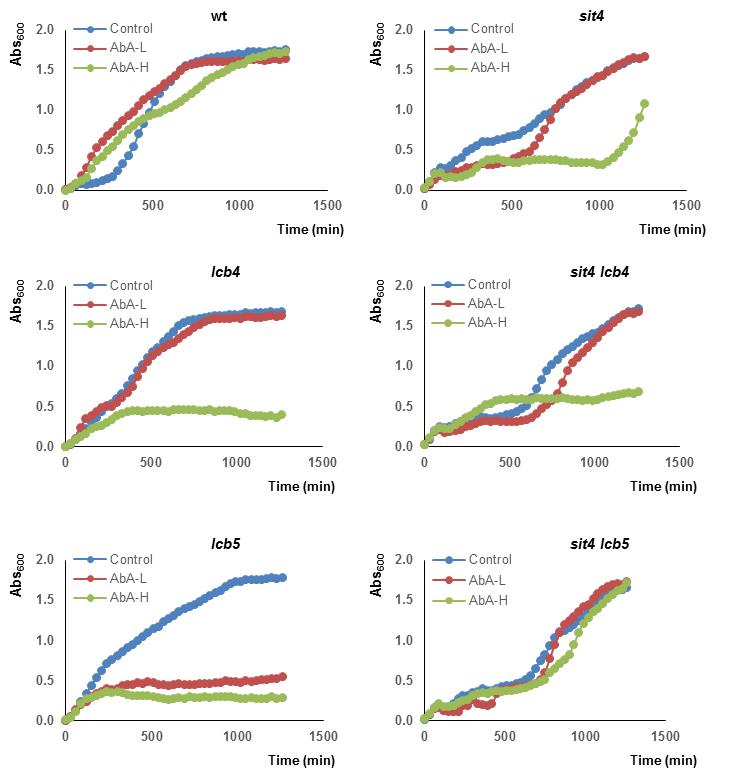


**FIGURE S2**. Sit4 shows a genetic interaction with Lcb4 and Lcb5. Saturated cultures of the indicated strains were diluted in fresh YPD-medium (Abs_600_ = 0.05) lacking (Control) or containing aureobasidin A at 0.034 μM (AbA-L) or 0.068 μM (AbA-H) and their growth was recorded by using a POLARstar Omega microplate reader. All assays were performed in triplicate at 30°C for 24 h.

**Bibliography cited**

Córcoles-Sáez I, Hernández ML, Martínez-Rivas JM, Prieto JA, Randez-Gil F (2016) Characterization of the *S. cerevisiae* *inp51* mutant links phosphatidylinositol 4,5-bisphosphate levels with lipid content, membrane fluidity and cold growth*. Biochim Biophys Acta* 1861: 213-226.

Prieto J, Estruch F, Córcoles-Sáez I, Rieger R, Del Poeta M, Stenzel I, Randez-Gil F. (2020) Pho85 and PI(4,5)P_2_ regulate different lipid metabolic pathways in response to cold. *Biochim Biophys Acta Mol Cell Biol Lipids* 1865: 158557.

Longtine MS, McKenzie A 3rd, Demarini DJ, Shah NG, Wach A, Brachat A, Philippsen P, Pringle JR (1998) Additional modules for versatile and economical PCR-based gene deletion and modification in *Saccharomyces cerevisiae*. *Yeast* 14: 953-961.

Goldstein A, McCusker J (1999) Three new dominant drug resistance cassettes for gene disruption in *Saccharomyces cerevisiae*. *Yeast* 15: 1541-1553.

Gietz R, Sugino A (1988) New yeast-Escherichia coli shuttle vectors constructed with in vitro mutagenized yeast genes lacking six-base pair restriction sites. *Gene* 74: 527-534.

Vandebroek T, Terwel D, Vanhelmont T, Gysemans M, Haesendonck CV, Engelborgh Y *et al*. (2006) Microtubule Binding and Clustering of Human Tau-4R and Tau-P301L Proteins Isolated from Yeast Deficient in Orthologues of Glycogen Synthase Kinase-3β or cdk5*. J Biol Chem* 281: 25388-25397.

Vanhelmont T, Vandebroek T, De Vos A, Terwel D, Lemaire K, Anandhakumar J *et al*. (2010) Serine^409^ phosphorylation and oxidative damage define aggregation of human protein tau in yeast. *FEMS Yeast Res* 10: 992-1005.
